# Immune Microenvironment of Brain Metastases—Are Microglia and Other Brain Macrophages Little Helpers?

**DOI:** 10.3389/fimmu.2019.01941

**Published:** 2019-08-20

**Authors:** Hua You, Szymon Baluszek, Bozena Kaminska

**Affiliations:** ^1^Affiliated Cancer Hospital & Institute of Guangzhou Medical University, Guangzhou, China; ^2^School of Laboratory Medicine, YouJiang Medical University for Nationalities, Baise, China; ^3^Affiliated Hospital of Academy of Military Medical Sciences, Beijing, China; ^4^Laboratory of Molecular Neurobiology, Nencki Institute of Experimental Biology, Warsaw, Poland

**Keywords:** neuro-oncology, brain metastases, tumor microenvironment, immune infiltrates, microglia

## Abstract

Brain metastases are common intracranial neoplasms and their frequency increases with prolonged survival of cancer patients. New pharmaceuticals targeting oncogenic kinases and immune checkpoint inhibitors augment both overall and progression-free survival in patients with brain metastases, but are not fully successful in reducing metastatic burden and still a majority of oncologic patients die due to dissemination of the disease. Despite therapy advancements, median survival of patients with brain metastases is several months, although it may vary in different types or subtypes of cancer. Contribution of the innate immune system to cancer progression is well established. Tumor-associated macrophages (TAMs), instead of launching antitumor responses, promote extracellular matrix degradation, secrete immunosuppressive cytokines, promote neoangiogenesis and tumor growth. While their roles as pro-tumorigenic cells facilitating tissue remodeling, invasion and metastasis is well documented, much less is known about the immune microenvironment of brain metastases and roles of specific immune cells in those processes. The central nervous system (CNS) is armed in resident myeloid cells: microglia and perivascular macrophages which colonize CNS in early development and maintain homeostasis in brain parenchyma and at brain-blood vessels interfaces. In this study we discuss available data on the immune composition of most common brain metastases, focusing on interactions between metastatic cancer cells and microglia, perivascular and meningeal macrophages. Cancer cells ‘highjack’ several CNS protective mechanisms and may employ microglia and CNS-border associated macrophages into helping cancer cells to colonize a pre-metastatic niche. We describe emerging molecular insights into mechanisms governing communication between microglia and metastatic cancer cells that culminate in activation of CNS resident microglia and trafficking of monocytic cells from the periphery. We present mechanisms controlling those processes in brain metastases and hypothesize on potential therapeutic approaches. In summary, microglia and non-parenchymal brain macrophages are involved in multiple stages of a metastatic disease and, unlike tumor cells, are genetically stable and predictable, which makes them an attractive target for anticancer therapies.

## Introduction

Cancer develops in a complex tissue microenvironment and non-malignant cells of a tumor microenvironment (TME) display tumor-promoting activities at many stages of cancer promotion and progression (1). Mutual communication between cancer cells and cellular components of microenvironment maintain normal tissue homeostasis, and supports tumor growth. This intercellular communication is operated by a multifaceted and dynamic network of cytokines, chemokines, growth factors, and enzymes remodeling extracellular matrix, leading to profound changes in properties of the surrounding tissue. Histopathological studies and flow cytometry analyses of human and rodent experimental tumors demonstrated cellular heterogeneity of a tumor niche and a vast infiltration of immune cells consisting several subpopulations: macrophages, granulocytes, myeloid-derived suppressor cells (MDSCs), and T lymphocytes. In a tumor microenvironment, immune cells are polarized into pro-invasive, immunosuppressive cells supporting tumor progression (1, 2). The signals they send lead to local and systemic immunosuppression which is difficult to overcome and could be a big obstacle in re-establishing antitumor immunity.

**Figure 1:**
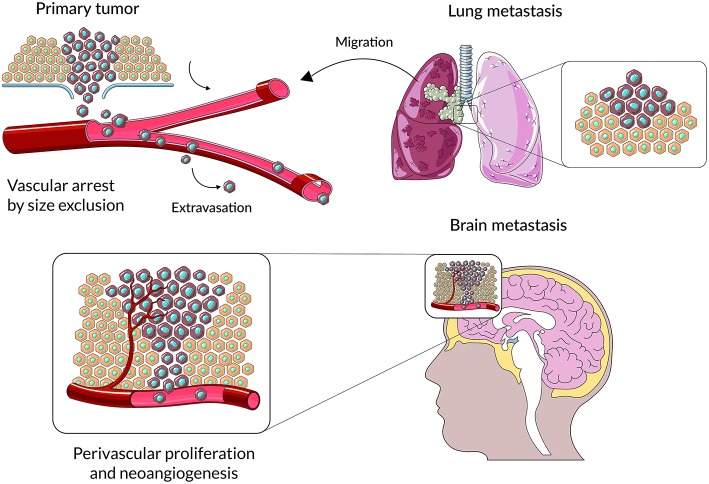
A schematic representation of main stages cancer cell colonization of brain parenchyma. Cancer cells from primary lesions invade into surrounding tissues, intravasate into the circulatory system and survive during hematogenous transit. Metastatic cells arrest at distinct sites and extravasate through vascular walls into the brain parenchyma. At the metastatic niche cancer cells proliferate, form colonies in this parenchyma; and the subsequent proliferation of cells leads to clinically detectable metastatic lesions.

Tumor-associated macrophages (TAMs) are found in many cancers and behaviors of the stromal cells within the tumor niche indicate that TAMs activate many processes related to wound healing and inflammatory reactions (2–4). TAMs have been divided into three main functional classes carrying out specific functions. Perivascular macrophages are localized along the vessels in the perivascular niche, where they support formation of intravasation sites where tumor cells spread into circulation and promote tumor angiogenesis (Figure 1). Streaming TAMs may co-migrate with tumor cells to a pre-metastatic niche, promote matrix remodeling, tumor invasion and form an immunosuppressive microenvironment. A third group of TAMs accumulate in a pre-metastatic niche and assist tumor cell extravasation, seeding, and formation of metastatic lesions. TAMs reorganize surrounding tissues, induce local and systemic immunosuppression and may help tumor cells to resist cytotoxic chemotherapy (5). Unlike tumor cells, stromal cells residing within TME are genetically stable, and thus exemplify an attractive target for therapeutic approaches, which is unlikely to exhibit drug resistance and contribute to tumor recurrence. Our knowledge regarding the composition and functions of specific cells within metastatic lesions is limited and even less is known about intracranial metastases.

Central nervous system (CNS) metastases, with incidence of 8.3 to 14.3 per 100,000 people (6), are recognized as the most common intracranial neoplasms. Their frequency increases with expanding life expectancy and prolonged survival of cancer patients. New pharmaceuticals targeting oncogenic kinases and immune checkpoint inhibitors increase overall and progression-free survival in patients with brain metastases, but do not reduce metastatic burden and still a majority of oncologic patients die due to dissemination of the disease. Despite therapy advancements, median survival of patients with brain metastases remains 6 months (7).

## Microglia are Immune Sentinels in the Central Nervous System

CNS is equipped in the resident, innate immune cells called microglia. Microglia originate from yolk sac myeloid progenitors, colonize CNS during early embryogenesis and persist throughout the entire life (8). Due to different ontogeny, location and highly specialized functions in a nervous tissue homeostasis and neuronal plasticity, microglia have a distinct transcriptional signature from peripheral macrophages (9). Under physiological conditions, microglia through numerous extension actively inspect the brain parenchyma and spinal cord, remove apoptotic debris by phagocytosis, participate in adequate tuning of neural circuits and without initation inflammation contribute to CNS homeostasis (10–13).

Microglia are sensors of any changes in CNS and rapidly react to any insult, infection or injury. Depending on activating stimuli, *in vitro* cultured microglia can display a spectrum of functional phenotypes with the extremes represented by an inflammatory phenotype associated with cytotoxicity or an opposite phenotype considered to be a pro-regenerative or pro-tumorigenic (14). Moreover, many neurological conditions are associated with infiltration of monocytes and other immune cells from the periphery. Recent studies using a single cell sequencing and cell lineage tracing demonstrated that resident microglia are functionally distinct from bone marrow-derived monocytes, which enter the CNS under pathological conditions (15, 16). For example, in malignant gliomas, common and diffusive brain tumors, microglia and peripheral macrophages are a main immune component of a tumor mass and their aberrant activation contributes to glioma progression by generating a hypoxic niche, which promotes genetic instability, supporting self-renewal of glioma initiating cells, instigating invasion, and calming anti-tumor immunity (16–18). Transcriptomic analysis and lineage tracing demonstrated distinct profiles in microglia and bone marrow (BM)-derived macrophages infiltrating murine gliomas and brain metastases (15). Based on those studies CD49D has been proposed as a good marker for flow cytometry to discriminate microglia and peripherally derived macrophages in human brain tumors (15). Markers, commonly used in immunohistochemistry, cannot distinguish resident microglia from invading monocytes in the human tissue, thus those cells are collectively called glioma-associated microglia and macrophages (GAMs). Tumor-activated GAMs release numerous factors facilitating invasion such as transforming growth factor β1, extracellular matrix digesting metalloproteinases (MMPs) and cathepsins. Several tumor-secreted factors such as osteopontin/SPP1 (19), versican (20), and periostin (21) have been reported as factors promoting re-education of microglia and macrophages infiltrating TME and therefore, shaping immune microenvironment of malignant gliomas. Both CNS resident microglia and peripheral macrophages bear respective integrin receptors binding osteopontin, periostin, or toll-like receptors (TLR) binding versican. Several other factors were indicated as chemoattractant and polarizing molecules. For example, colony stimulating factors (CSFs) are chemoattractants for microglia and monocytes, and polarize those cells into a pro-tumorigenic phenotype. CCL2 (a chemokine (C-C motif) ligand 2, known previously as MCP-1) is released from human glioma cells and attracts microglia expressing a receptor CCR2 (18).

**Figure 2:**
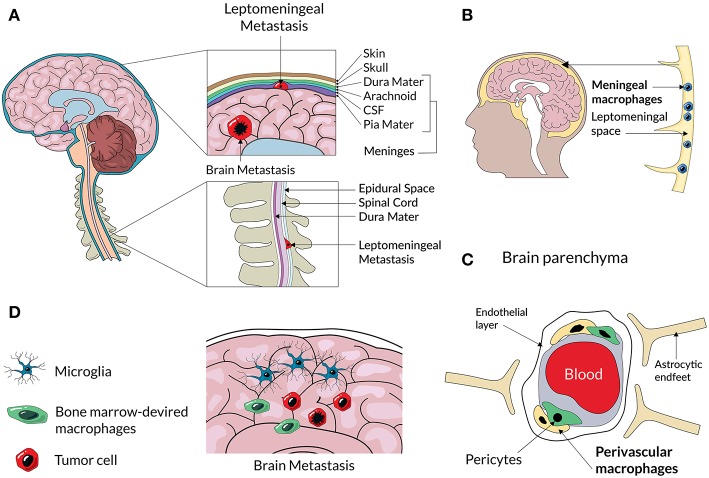
A scheme representing an anatomical location of different CNS macrophage populations and CNS metastases. Cancer cells from primary tumors enter to the brain and cerebrospinal fluid (CSF) and can form secondary tumors within the brain parenchyma, or and spread to the membranes of leptomeninges surrounding the brain and spine **(A)**. Non-parenchymal macrophages located at the meninges **(B)** and perivascular spaces **(C)** can assist extravasation and spread of metastatic cancer cells. Metastatic lesions in the brain parenchyma consist of resident microglia and infiltrating BM-derived macrophages **(D)** that create a metastatic niche.

Colonization of the CNS by cancer cells from a periphery and interactions of invading cells with the host tissue are still poorly understood. CNS colonization by metastatic cells from a periphery is associated with complex processes such as extravasation from blood vessels, tissue remodeling and death of neurons (22). The latter may be recognized as disturbance of CNS homeostasis and elicit recuperating responses from microglia to protect, repair, and instigate the wound healing, associated with local immunosuppression. All these processes are actively assisted by microglia and infiltrating peripheral macrophages through mechanisms that are poorly characterized (23). One of the unanswered questions is whether responses of microglia to infiltrating metastatic cancer cells are tumor-type specific or more alike, and whether microglia (and non-parenchymal macrophages) play specialized roles, as peripheral TAMs. CNS is equipped in specialized macrophages such perivascular, meningeal and choroid plexus macrophages (Figure 2). While those cells have different transcriptomic signatures and location from microglia (those cells are non-parenchymal macrophages), they colonize CNS at the same time as microglia and generally, do not exchange with peripheral macrophages (8, 14). Due to their properties and location at the blood vessels-CNS interfaces, those cells may actively assist cancer cells to extravasate to brain parenchyma. Here, we attempt to fill this gap in knowledge by discussing recent insights about heterogeneity of immune infiltrates in the microenvironment of common CNS metastases, potential factors, and mechanisms responsible for polarization of immune cells induced by cancer cells and their mutual interactions.

## Brief Characteristics of CNS Metastases

CNS metastases are found in autopsies of 20% cancer patients (24). The clinically reported incidences vary depending on methodology, but the estimated incidence of CNS metastases is 9% of all cancer patients (25, 26), more if the disease was primarily disseminated (27). The incidence varies between different tumor types and spans between 40 and 50% for lung cancer, 20 and 30% for breast cancer, 20 and 25% for melanoma, 10 and 20% for renal carcinoma, and 4 and 6% for gastrointestinal tumors. CNS metastases develop predominantly within the brain parenchyma, and leptomeninges. The leptomeninges are composed of three layers of a fibrous tissue known as the dura mater (the layer lying directly beneath the skull), arachnoid, and pia mater (28). Cerebrospinal fluid fills brain ventricles, the space between the pia mater and the arachnoid. Those predominant locations of CNS metastasis are depicted in the Figure 2A. The incidence of CNS metastases is increasing, likely due to increasing age population and prolonged survival of patients with primary and secondary advanced cancers (29). Moreover, advancements in imaging techniques result in detecting metastases more effectively (30).

CNS metastases are a main cause of illness, affect cognitive functions, speech, coordination, behavior, reduce a quality of life and ultimately lead to death. The standard of care in CNS metastases includes local treatment with surgery, stereotactic radiosurgery or stereotactic fractionated radiotherapy combined with systemic chemotherapy. Despite advancements in treating patients with metastatic cancer (31, 32), a metastatic burden is a cause of death in 90% of cancer patients (33). With modern treatment the median survival with CNS metastases is 6 months (34), but the results vary depending on tumor histology, disease control, patient age, and initial therapy responses (35). CNS metastases typically have a poor prognosis and result in shortening survival of patients to 3–6 months, so patients suffering from them are often excluded or underrepresented in clinical trials of new pharmaceuticals. For instance, in melanomas inhibitors of mutant BRAF or immune checkpoint inhibitors did not decrease frequency of developing *de novo* CNS metastases, but improved patient survival. Clinical trials in melanoma brain metastases with combined PD-1/CTLA-4 blockade showed ~50% intracranial response rate (36–38). Moreover, checkpoint inhibitors are considered effective as radiosensitizers in CNS metastases (39).

Metastases are relatively uncommon in children. The autopsy studies estimated frequency of CNS metastasis at 13% (40) and at 1.5% in clinical studies (41). CNS involvement is common in pediatric leukemias and rare in solid tumors, where the occurrence is highest in germ cell tumors, bone and soft tissue sarcomas (6, 41). For leukemias and lymphomas, CNS invasion is carried by blood and spreads via the arterial and capillary system, or via direct expansion from the cranial bone marrow. The choroid plexus (a tissue with a vast network of capillaries) and bridging veins between the bone marrow and superficial arachnoid are assumed to be sites of cancer cell invasion into the CNS (Figure 2A).

Most CNS metastases originate from lung and breast cancers, and melanoma (25.8, 22.4, and 18.2%, respectively) (42). Those three cancers bear the highest risk of metastasizing to CNS (24, 26). Most patients with CNS metastases have simultaneously non-cerebral metastases. Comparative studies from The Cancer Genome Atlas (TCGA) showed that in 11 studied cancer types some differential expression patterns associated with metastasis were shared across multiple cancer types, but in fact each cancer type showed a distinctive metastasis signature. Functional categories of genes enriched in a common metastasis signature included cellular response to stress, DNA repair, oxidation-reduction process, protein deubiquitylation, and receptor activity (43). A recent TCGA study interrogating genomics of a tumor-of-origin and its metastasis among thousands samples of 33 tumor types revealed that metastases retained the mutational landscape of tumor of origin (44).

In this study we discuss published data on immune components of CNS metastases with a focus on microglia (and other brain macrophages) and their roles in shaping metastatic niches. Several studies reported the accumulation of HLA-DR+ microglia/macrophages in the intracranial metastatic lesions in breast, melanoma, small cell lung, and non-small cell lung cancers (45), but understanding which roles they play in metastatic seeding of CNS and lesion progression escaped prior notice.

## Immune Microenvironment of Lung Cancer Metastases

Lung cancer cells most frequently form CNS metastases and patients with such disease have a poor prognosis, a median survival is 6–7 months (46). Non-small-cell lung cancer (NSCLC) is most common in invading CNS (70% of metastases affect brain) and many patients with stage III or IV cancer (up to 55%) develop CNS metastases in a course of the disease (47). A fraction of patients (3–5%) develops leptomeningeal metastases (48). A histological subgroup of small-cell lung cancer (SCLC) affects 20% of patients and is particularly aggressive. CNS metastases are treated with carboplatin, etoposide, and preventive cranial irradiation (49). Despite recent advancement with anti-PD1 antibody atezolizumab, a patient's prognosis remains poor (50). EGFR (epidermal growth factor receptor) inhibitors are recommended as a therapeutic option because EGFR-positive NSCLC metastases to CNS occur more often than other subtypes (51). A recent trial with osimertinib, 3rd generation EGFR inhibitor showed superiority over previous generation EGFR inhibitors, also in CNS metastases (52). NSCLC responds relatively well to immunotherapy. NSCL patients without *EGFR* mutations and with expression of PD-L1 on ≥50% of tumor cells reacted better to the treatment with pembrolizumab (anti-PD-1 antibody) than to platinum chemotherapy, both in terms of overall survival and side-effect profiles (53). Similar results were obtained in the trial comparing anti-PD1 antibody nivolumab and paclitaxel in squamous-cell NSCLC (54).

One of a few studies exploring the occurrence of brain macrophages in human CNS metastases used the anti-CD68 antibody on paraffin-embedded tissue specimens of 17 metastatic tumors including: 7 lung carcinomas, 5 breast carcinomas, 3 clear cell kidney carcinomas. CD68+ macrophages were localized within the tumor tissue, at its periphery and its surroundings. In some cases, cells with an intensive staining reaction were visible in blood vessel walls. No correlation between the type of tumor and intensity of macrophage infiltration has been found (55). Unfortunately, the immune composition of CNS metastases of lung cancer has not yet been explored and there is no more specific data on immune populations.

In lung cancer CNS metastatic lesions, Iba1+ cells were found close to the cancer cells, and showed amoeboid, activated morphology. Double labeling revealed that a majority of Iba1+ cells do not co-localize with either iNOS or TNF-α staining, which suggests their deficits of anti-tumor responses and a pro-tumorigenic phenotype (56). Another study reported the high number of CD68+ cells in metastases of adenocarcinoma and most positive cells displayed amoeboid morphology. The study did not show consistent differences between benign and malignant neoplasms (this was likely due to a small number of samples) (57). Even those fragmentary studies suggest the potential role of microglia and/or macrophages in metastatic lesions.

The analysis of subsets of tumor infiltrating lymphocytes (TILs) representing main types of cells such as CD3+, CD8+, CD45RO+, FOXP3+ and PD-1+, and the expression of PD-L1 in SCLC brain metastases and four matched primary tumor specimens using immunohistochemistry showed the active immune microenvironment in SCLC brain metastases. PD-L1 was expressed on TILs and on tumor infiltrating macrophages. Patients with higher numbers of infiltrating CD45RO+ TILS survived longer (58). Similar proportions of TILs were detected in NSCLC (59). The observation that TIL infiltration is irrespective of tumor stage (60) suggests an active immune microenvironment in brain metastasis and provides an encouragement to apply the immune check point inhibitors also in a therapy of patients with lung cancer CNS metastases.

## Immune Microenvironment of Breast Cancer Metastases

Molecular classification of breast cancer relies on the expression of estrogen receptor (ER), progesterone receptor (PR) and human epidermal growth factor receptor-2 (HER-2). The triple-negative breast cancer occurs in 15–20 % of patients and is its most aggressive form. Its metastases to the CNS are clinically discovered in 46% of patients, but this seems to be due to overall metastasis burden rather than a specific property of this molecular subgroup (61). HER-2 positive breast cancer, found in 15–20% of patients, has better prognosis and responds to trastuzumab. CNS metastases are detected in up to 50% patients and can develop years after initial diagnosis, likely because trastuzumab does not cross blood-brain barrier (BBB) (62). Lapatinib, a novel inhibitor targeting a tyrosine kinase, inhibits both HER-2 and EGFR, and crosses BBB. Recently, a meta-analysis of phase II trials combining lapatinib with capecitabine in HER-2 positive breast cancer patients and brain metastases recommended this therapy as a first- or second-line treatment (63). In general, CNS metastases of breast cancer prognosticate better than to other sites (13.8 months) and respond to a systemic therapy in up to 80% of cases (46). Staining with the microglia/macrophage marker KiM1P in human breast cancer metastases to CNS showed positive cells in all samples and accumulation of activated (non-ramified), positively stained cells in the boundary region between tumor and neighboring tissue. Their amount varied from only few up to 50% of all cells (64).

CNS metastasis of breast cancer is modeled by injection of cancer cells via a carotid artery to immunodeficient or immunocompetent mice. While cancer cells need more time to extravasate into the brain parenchyma than into other organs, human breast cancer cells of different lines MDA-MB-435, MDA-MB-231/brain, and 4T1 form tumors in immunosuppressed SCID mice. Using an animal model allowed to study the dynamics of invasion and demonstrated that cancer cells are halted within the brain microvessels and extravasate from day 3 to day 7, with exception of MDA-MB-231/brain cells, which were slower (65). Cells invading the brain parenchyma induced a local activation of astrocytes and microglia. Astrocytes up-regulated expression of GFAP (glial fibrillary acidic protein), Nestin, or both. Microglia (detected with F4/80 staining) infiltrated into the breast cancer mass, accumulated in the surrounding gliosis zone, and formed contacts with tumor cells directly after successful extravasation (65, 66). Microglia associated with cancer cells were heterogeneous and consisted of activated, hypertrophic microglia and reactive microglia with amoeboid cell morphology (65). Using an intracardiac injection of 99LN-BrM cells derived from MMTV:PyMT breast cancer cells to syngeneic, immunocompetent mice with Cx3cr1-based myeloid cells (a lineage tracing model), the authors found both microglia and infiltrating BM-derived macrophages in brain metastases (15).

*In vitro* studies on microglia co-cultured with different breast cancer cells (MCF-7 and 410.4) and in living brain slice cultures (in which peripheral blood-derived macrophages were absent) showed that microglia promote cancer cell invasion and colonization of the brain tissue. Blocking microglia function with the bisphosphonate clodronate reduced cancer cell invasion. Stimulation of the TLR4 pathway shifted microglia to a pro-inflammatory and anti-invasive phenotype. In organotypic brain slice cultures microglia (stained with isolectin B4, ILB4) helped to transport a single invading cell as well as cancer cell cohorts. Gene expression studies of microglia co-cultured with carcinoma cells did not show up-regulation of the pro-tumorigenic (M2) phenotype gene markers, but identified TLR and WNT signaling as the most affected pathways in those microglia. Both pathways were previously implicated in controlling tissue regeneration and repair. Microglia-induced invasion in Boyden chamber co-cultures and in living brain slices was completely abolished by DKK-2 (a secreted Wnt antagonist known to antagonize predominantly Wnt/β-catenin signaling) (64). The *cxcr4* gene coding for C-X-C chemokine receptor 4 (CXCR4) was one of the most up-regulated genes in microglia (64). CXCR4 and its ligand stroma derived factor 1 (SDF1) are up-regulated in various cancers, and CXCR4 inhibition prevented metastasis formation (67). Studies of human breast cancer MCF-7 cells and the benign Madin–Darby canine kidney cells (MDCK) tested in brain slice cultures demonstrated that microglia support invasion of breast cancer MCF-7 cells, but not the benign epithelial MDCK cells. The WNT-inhibitor DKK2 as well as a CXCR4 inhibitor—AMD3100, reduced invasion of MCF-7 into the whole brain slice to similar extent (68).

CD11b+F4/80+ cells were detected by flow cytometry as the most abundant infiltrating immune cell population in brain metastases developing after intracarotid injection of different breast cancer cells (4T1, PyMT, or MDA-MB-231). The infiltration of myeloid-derived suppressor cells (MDSCs; CD11b+Gr1+), granulocytes (CD11b+Ly6G+) and monocytes (CD11b+Ly6C+) into dural metastases was greater than in parenchymal lesions (69). Only a very low infiltration rate of T-cells (CD3e+) was detectable at either location. Interestingly, gene expression profiling revealed significant differences in gene expression of cancer cells that have metastasized to the brain parenchyma or the dura, with the high level of mRNA for Lymphotoxin β (LTβ) in parenchymal vs. dural metastatic lesions. The lower levels of *inos, MHCII, CD11c, arg1, ifn*γ *and tnd*α in CD11b+F4/80+CD45^high^ cell population (bone marrow-derived macrophages) from parenchymal metastasis were detected. The expression of *cd206* (a pro-tumorigenic phenotype marker) was significantly increased in CD11b+F4/80+CD45^high^ cells parenchymal lesions. This pattern of marker gene expression suggests that the parenchymal macrophages are more twisted toward the pro-tumorigenic phenotype compared to dural cells. It also confirms that a location of a metastatic site matters (69).

Accumulation of GAMs was found around mammary 4T1-GFP carcinoma cells intracranially implanted to Balb/c mice (at different times after implantation). A strong positive correlation was found between the Iba1 immunostained area and the volume of the 4T1-GFP metastases. The expression of pro-tumorigenic markers: arginase1 (Arg1) and mannose receptor 1 (Mrc1), as well as the pro-inflammatory markers such as inducible nitric oxide synthase (iNos) and cyclooxygenase 2 (Cox2) was detected around those tumor loci. Depletion of MRC1+ microglia/macrophages in the 4T1-GFP metastatic brain by intracerebral injection of mannosylated clodronate liposomes significantly reduced metastasis growth in the brain (70). Systemic treatment of 4T1-bearing mice with anti-Gr1 (RB6-8C5) monoclonal antibody reduced accumulation of CD11b+Gr1+ myeloid cells in the pre-metastatic brain and subsequent brain metastases of 4T1 cells. Treatment of 4T1 tumor-bearing mice with the Cox-2 inhibitor celecoxib or knockdown of Cox-2 in 4T1 cells inhibited up-regulation of inflammatory chemokines and infiltration of CD11b+Gr1+ myeloid cells in the pre-metastatic niche and subsequent formation of brain metastasis. Intraperitoneal administration of anti-CCL2 mAb reduced percentages of CD11b+ cells and expression of S100A8, S100A9, and SAA3 in the brain of BALB/c mice bearing 4T1 tumors (71). Inhibition of CCL2-CCR2 signaling blocked the recruitment of activated monocytes, inhibited metastasis *in vivo* and prolonged the survival of tumor-bearing mice. Depletion of tumor-cell-derived CCL2 also inhibited metastatic seeding (72). These data strongly support a critical role of microglia in metastasis of mammary carcinoma cells to brain parenchyma.

The studies with inhibitors of specific signaling pathways discussed above identified Wnt/β-catenin, CXCR4-SDF1 and CCL2-CCR2 signaling pathways as crucial for microglia-cancer cell communication in breast cancer CNS metastases (64, 68). Furthermore, PI3K signaling was found active in the majority of breast cancer brain metastases. A systematic quantification of the PI3K pathway activity in breast cancer CNS metastases, using a reverse phase protein array, found a high PI3K signaling activity in 62.5% brain metastatic tissues. PI3K signaling was activated in metastasis-promoting microglia/macrophages during CNS colonization. Treatment with a pan-PI3K Class I inhibitor - buparlisib (BKM120), reduced their metastasis-promoting activity (73).

## Immune Microenvironment of Melanoma Metastases

Metastases to the CNS were found in 10–40% of melanoma patients, although the higher number of metastatic lesions 70– 90% were detected in brains post mortem. Melanoma cells show some preferences as to location in CNS. Investigation of 115 brain metastases revealed that the majority was located within the frontal lobe (43.5%), less frequently in the cerebellum (8.6%) and rarely found in the hippocampus (<0.1%) (74). *BRAF* mutations occur in 40–50% of melanomas and treatments with specific inhibitors (e.g., vemurafenib, dabraenib) were reported to be effective in a metastatic disease. The presence of *BRAF* mutation does not affect probability of CNS metastases, but a targeted treatment with vemurafenib decreases such probability (75). Addition of a MEK inhibitor trametinib in a phase II trial was reported to be more effective (76). Moreover, melanomas are highly immunogenic tumors and checkpoint inhibitors have been very successful, with pembrolizumab superior to ipilimumab (which targets CTLA-4). Combination of nivolumab with ipilimumab was superior to ipilimumab alone (77). Significant improvements have extended the therapeutic options for treating brain metastases from melanoma, by combining potent BRAF inhibitors with checkpoint inhibitors or stereotactic surgery (78).

Administration of cancer cells via an internal carotid artery has been used a model to test the potential of two human melanoma cell lines for organ colonization in three immunodeficient mouse strains: nude (nu/nu), NIH triple immunodeficient (TID: nu/nu, bg/bg, xid/xid) and severe combined immunodeficient (SCID) mice. Studies revealed that MM-RU melanoma cells gave rise exclusively to lung metastases, whereas the MM-AN cells gave rise to lung and extra-pulmonary metastases. The metastatic lesions were circumscribed in all organs and exhibited peripherally located macrophages, except for brain metastases, where a more invasive pattern along vasculature was observed (79).

Further studies provided results suggesting some degree of specificity in CNS colonization. K-1735 melanoma cells injected into the internal carotid artery of mice produced metastatic lesions only in the brain parenchyma, whereas B16 melanoma cells and mixed B16 x K-1735 melanoma cells produced metastatic lesions only in the leptomeninges and ventricles. This difference in metastatic tumor location was attributed to the expression of transforming growth factor-beta 2 (TGF-β2) in cancer cells: *TGF-*β*2* mRNA was highly expressed by the K-1735 cells, whereas the B16 cells or B16 x K-1735 cell mixes had low expression. Manipulation of TGF-β2 expression in melanoma cells reduced metastasis to the brain parenchyma, but did not affect metastasis to the leptomeninges or ventricles (80).

In a transplantable model of spontaneous melanoma brain metastasis in immunocompetent mice activated astrocytes and microglia (stained with isolectin B4, ILB4) were recruited to the tumor–brain interface (81). The dynamic changes of microglia and macrophages during formation of brain metastasis following intracranial melanoma cell implantation were visualized through long-term intravital imaging using CX3CR1-GFP transgenic mice. Depletion of microglia and macrophages by treatment with PLX3397, an inhibitor of colony stimulating factor-1 receptor (CSF-1R), reduced the total number and mean size of the brain metastases by 83 and 65%, respectively. Microglia and macrophages from metastatic brains expressed MMP3 and treatment with PD166793, an MMP inhibitor, reduced the total number and mean size of the brain metastases by 50 and 53%, respectively (82). In a transplantable model of B16 melanoma cells the presence of microglia (CD11b+F4/80+CD45^low^) infiltration into intracranial B16 melanoma tumors increased following combined PD-1/CTLA-4 blockade and the increase in microglia correlated with intracranial therapeutic efficacy. Simultaneous increase in CSF-1 within tumors was observed, potentially explaining increased microglia infiltration (36). The results point to supporting role of tumor infiltrating microglia and macrophages in the melanoma CNS metastases.

## Meningeal Metastases

The dura mater is the outermost of the three layers of meninges (Figures 2A,B). Metastases to the dura (pachymeningeal metastases) were found during autopsy in 9–10% of all patients with cancer and are the solely site of intracranial metastases in about 4% of patients (83). Neoplastic spread to leptomeninges is a result of cancer dissemination to the cerebrospinal fluid. It occurs when cancer cells gain access to cerebrospinal fluid pathways, travel to multiple sites within the central nervous system, colonize, and grow. Leptomeningeal metastases occur in 1–5% of patients with solid tumors (and are also called carcinomatous meningitis), in 5–15% of patients with leukemia (leukemic meningitis) and in 1–2% of patients with primary brain tumors (84). Melanoma, lung and breast cancers have highest potential of causing carcinomatous meningitis and a median survival is extremely short (2 months). Survival has been improved by a systemic chemotherapy and whole-brain radiotherapy (85) and treatments with new chemotherapeutics brought improvements in cases with melanoma and lung cancer metastases (86). While there is no data on detection microglia and macrophages is those tumors, the increasing knowledge about specific populations of perivascular and meningeal macrophages prompts us to speculate on a potential role in those macrophages in CNS metastases.

Non-parenchymal macrophages (expressing CD11b and CD163 antigens) reside in perivascular and leptomeningeal spaces, and the choroid plexus (87) (Figures 2B,C). Perivascular macrophages are elongated along the blood vessels and form close contacts with endothelial cells. Like microglia, they originate from a yolk sac and persist through life, with the exception of the choroid plexus where a minor exchange with blood monocytes takes place (87). These cells are characterized by the CX3CR1+CD11b+CD45^high^ profile in flow cytometry. Due to location at the blood-brain interfaces perivascular macrophages are involved in immune-surveillance and establish a gateway for the recruitment of peripheral immune cells into the CNS in a response to pathological stimuli (88). Location of leptomeningeal metastases may be indicative of a supportive role of leptomeningeal macrophages in extravasation and seeding of tumor cells in the CNS parenchyma. Their exact roles of CNS cancer metastases should be elucidated.

## Microenvironment of Haematopoietic CNS Metastases

Leptomeningeal, epidural and brain parenchyma metastases are the most common neurologic complications of non-Hodgkin's lymphomas and are associated with a poor prognosis (89). The information regarding immune infiltrates in those metastases is lacking. Acute lymphoblastic leukemia (ALL) has a marked tendency to metastasize to the CNS, occurs in 5% of patients and ALL relapse in the CNS predicts poor outcomes. CNS-directed therapies such as: cranial irradiation, intrathecal chemotherapy and systemic administration of CNS-penetrating chemotherapeutics, have reduced the frequency of disease recurrence (90). Spread of ALL rarely involves the parenchyma, but is localized to the leptomeninges.

Xenotransplantation of human ALL cells in immunodeficient NSG mice provokes a disease with neurologic symptoms characterized by the infiltration of leukemic cells entirely restricted to meninges without any direct involvement or infiltration of the parenchyma. CXCR4 inhibition (with CXCR4 antagonist AMD-3100) impaired grafting of T-ALL cells to bone marrow, leukemia development and CNS infiltration (91). A recent study showed that ALL cells migrate into the CNS along vessels that pass directly between the bone marrow and the subarachnoid space. By traveling along the external surface of vessels that are topologically connected with the CNS subarachnoid space, ALL cells migrate directly from the bone marrow to the CNS, evading the necessity to enter and exit the CNS vasculature. The basement membrane of these vessels is enriched in Laminin and the Laminin receptor α6 integrin is expressed in most cases of ALL. Interactions of α6 Integrin-Laminin mediated the migration of ALL cells toward the cerebrospinal fluid *in vitro*. Mice with ALL xenografts treated with a PI3Kδ inhibitor (which decreased α6 integrin expression on ALL cells) or specific α6 integrin-neutralizing antibodies, showed significant reductions in ALL transportation along vessels (92).

## Microglia and Macrophages in Support of Primary Central Nervous System Lymphoma

Primary central nervous system lymphoma (PCNSL) is a primary tumor but due to its location in CNS and interactions with local immune cells shares certain mechanisms with CNS metastases. PCNSL is a rare form of lymphoma which accounts for 3–4% of all primary brain tumors and 4–6% of extra-nodal lymphomas (93). The majority of PCNSL is pathologically classified as diffuse large B-cell lymphoma (DLBCL) confined to CNS. The question whether B cells home to the CNS in a benign or malignant state has not been definitely answered. B cells which have been recruited to the CNS in the course of an immune reaction, most likely in response to a pathogen, could persist for extended time and eventually transform while residing in the brain. On the other hand, B cells could be transformed outside the CNS and entered the CNS thereafter. Standard chemotherapeutic regimens for systemic DLBCL show a little efficacy in PCNSL, likely due to inefficient drug delivery across the blood-brain barrier (94). High-dose methotrexate in combination with cytarabine is currently used as a treatment, but such chemotherapy with or without whole-brain irradiation is associated with neurotoxicity, and eventual relapse of lymphoma is frequently observed. The prognosis of PCNSL is poor with a median overall survival of 1–4 years, and it becomes even shorter in immunocompromised patients (93, 94). The most comprehensive genomic study of PCNSL samples using whole-exome sequencing and RNA-sequencing revealed that PCNSL and DLBCL share some common gene expression and mutation profiles (94). However, bioinformatic analysis showed a distinct DNA methylation profile of PCNSL from systemic DLBCLs or normal lymph nodes, with a subset of systemic DLBCL sharing a similar methylation profile with PCNSL. These genetic (94) and epigenetic (95) studies suggest that PCNSL is a biologically distinct entity from peripheral DLBCLs.

There is a little information regarding microglia/macrophages in PCNSL, partly due to its rarity and the lack of tissue specimens as most cases are diagnosed by stereotactic biopsy. The first study assessing the phenotypes of myeloid cells was performed with a small PCNSL cohort (*n* = 43), and numbers of CD68+, CD163+, and CD204+ TAMs were not associated with prognosis of patients (96). In another study of independent PCNSL cohort (*n* = 47), contrary to their conclusion, increased numbers of CD68+ TAMs were significantly associated with progression-free survival, and a trend was observed for the increased CD163+ TAMs and shorter survival. Increased TAMs were not associated with overall survival. Other factors such as: cerebrospinal fluid (CSF) interleukin-6 (IL-6) levels and soluble IL-2 receptor were not correlated with TAMs infiltration. The CSF IL-10 level was correlated with infiltration of CD68 and CD163+ TAMs (97), and the diagnostic and prognostic biomarker value of IL10 in the cerebrospinal fluid in PCNSL was confirmed in a series of studies (98–100). The recent study including the largest PCNSL cohort (*n* = 114) showed that the numbers of tumor-infiltrating immune cells affected the clinical outcome of patients with PCNSL. Briefly, the increased number of CD68+ TAMs and the increased number of indoleamine 2,3-dioxygenase (IDO) positive cells were associated with a favorable prognosis, whereas the increased number of CD204+ cells and a high ratio of CD204+/CD68+ cells, indicative of M2-like polarization, were associated with a poor prognosis in PCNSL (101). *PD-L1* and *IDO1* were overexpressed by macrophage/microglia in PCNSL tissues, and gene expression profiling indicated that *IDO1* expression was positively correlated with the expression of macrophage and lymphocyte markers (102). The expression levels of *CCL2* (MCP-1) mRNA and CCL2 protein were significantly increased in PCNSL compared with DLBCL. Stimulation of a human brain-derived lymphoma HKBML cells with CCL2 induced tyrosine phosphorylation of mitogen-activated protein kinase (103).

Interestingly, *SPP1* (coding for a small phosphoprotein Osteopontin being a strong inducer of microglia and other immune cells) is the most upregulated gene in PCNSL compared to non-CNS DLBCL (102, 104). Overexpression of Osteopotin up-regulates invasiveness of B lymphoma cells in murine brain slices, promotes intracerebral invasion and dissemination of lymphoma cells. It increases the intracerebral lymphoma growth and shortens the survival in athymic mice. Mechanistically, these effects depend on intracellular Osteopontin, which acts on transcription factor NFκB and causes transcriptional downregulation of the NF-κB inhibitors, *A20/TNFAIP3 and ABIN1/TNIP1*, and secretory Osteopontin which promotes receptor-mediated activation of NF-κB (102). Preclinical animal models mimic well the clinical course and neuropathology of human PCNSL and show pathological interactions between the malignant B cells, resident cell populations of the CNS, and the associated immune infiltrates. The preferential residence of PCNSL cells in the perivascular space may indicate interactions of the malignant B cells with components of the blood–brain barrier: endothelial cells with upregulated MHC class I and II antigens, ICAM-1, and vCAM-1, and perivascular macrophages. Also activation of resident brain cells was confined to areas of lymphoma infiltration, where microglia upregulated ICAM-1, MHC class I and II antigens, and astrocytes upregulated GFAP (105).

The discovery of immunotherapy targeting the programmed death-1 (PD-1, CD279)/its ligand (PD-L1, CD274) immune checkpoint pathway or cytotoxic T lymphocyte antigen-4 (CTLA-4) has emerged as potent and effective therapy for PCNSL patients. PCNSL frequently exhibited 9p24.1/ PD-L1/PD-L2 copy number alterations and translocations, which could be genetic bases of immune evasion (106). Treatment with nivolumab, an antagonistic antibody for PD-1, resulted in a significantly higher rate of complete remission for refractory PCNSL patients (107). However, the functional roles of PD-L1 may be opposite on lymphoma cells and/or on TAMs in PCNSL. In a PCNSL cohort (n = 64), lymphoma cell PD-L1 expression correlated positively with overall survival, whereas PD-L1 expression in the microenvironment exhibited a negative trend with overall survival (108).

## Conclusions

Breast, lung, and melanoma cancer cells have high propensity to migrate toward the brain. The brain parenchyma and the leptomeninges/ventricular system represent two distinct microenvironments in the CNS and certain cancer cells preferentially colonize those sites acquiring different features in the process. Survival and seeding to a new niche in CNS are supported by several mechanisms in which different brain cells take part, with a prominent role of microglia (and potentially non-parenchymal macrophages). Cancer cells take-over several mechanisms and polarize microglia and infiltrating peripheral macrophages, which in turn increases proliferation and survival of cancer cells (109). Growing evidence points to a significant and unexplored role of microglia and non-parenchymal macrophages in CNS metastases. There are several good reasons for the limited number of reports on microglia-metastatic carcinoma interactions: a difficulty of distinguishing resident microglia from invading bone marrow-derived monocytes/macrophages due to a lack of convenient markers for immunohistochemistry, the limited availability of patient samples, and the lack or shortage of suitable mouse models to study the brain microenvironment during CNS colonization by metastatic cancer cells. Better understanding of those interactions is necessary as there is a growing number of therapies specifically targeting tumor infiltrating microglia/macrophages that could be used in therapy of CNS metastasis.

## Author Contributions

HY, SB, and BK conceived the hypothesis, did the literature search, and wrote the manuscript. BK prepared the figures.

### Conflict of Interest Statement

The authors declare that the research was conducted in the absence of any commercial or financial relationships that could be construed as a potential conflict of interest.

## References

[1] Hanahan D Coussens LM. Accessories to the crime: functions of cells recruited to the tumor microenvironment. Cancer Cell. (2012) 21:309–22. 10.1016/j.ccr.2012.02.022 22439926

[2] Grivennikov SI Greten FR Karin M. Immunity, inflammation, and cancer. Cell. (2010) 140:883–99. 10.1016/j.cell.2010.01.025 20303878 PMC2866629

[3] Muliaditan T Caron J Okesola M Opzoomer JW Kosti P Georgouli M. Macrophages are exploited from an innate wound healing response to facilitate cancer metastasis. Nat Commun. (2018) 9:2951. 10.1038/s41467-018-05346-7 30054470 PMC6063977

[4] Mantovani A Allavena P Sica A Balkwill F. Cancer-related inflammation. Nature. (2008) 454:436–44. 10.1038/nature07205 18650914

[5] Sanchez LR Borriello L Entenberg D Condeelis JS Oktay MH Karagiannis GS. The emerging roles of macrophages in cancer metastasis and response to chemotherapy. J Leukoc Biol. (2019) 106:259–74. 10.1002/JLB.MR0218-056RR 30720887 PMC6779158

[6] Nayak L Lee EQ Wen PY. Epidemiology of brain metastases. Curr Oncol Rep. (2012) 14:48–54. 10.1007/s11912-011-0203-y 22012633

[7] Smedby KE Brandt L Bäcklund ML Blomqvist P. Brain metastases admissions in Sweden between 1987 and 2006. Br J Cancer. (2009) 101:1919–24. 10.1038/sj.bjc.6605373 19826419 PMC2788258

[8] Prinz M Priller J. Microglia and brain macrophages in the molecular age: from origin to neuropsychiatric disease. Nat Rev Neurosci. (2014) 15:300–12. 10.1038/nrn3722 24713688

[9] Butovsky O Jedrychowski MP Moore CS Cialic R Lanser AJ Gabriely G. Identification of a unique TGF-β-dependent molecular and functional signature in microglia. Nat Neurosci. (2014) 17:131–43. 10.1038/nn.3599 24316888 PMC4066672

[10] Nimmerjahn A Kirchhoff F Helmchen F. Resting microglial cells are highly dynamic surveillants of brain parenchyma *in vivo*. Science. (2005) 308:1314–8. 10.1126/science.1110647 15831717

[11] Glass CK Saijo K Winner B Marchetto MC Gage FH. Mechanisms underlying inflammation in neurodegeneration. Cell. (2010) 140:918–34. 10.1016/j.cell.2010.02.016 20303880 PMC2873093

[12] Miyamoto A Wake H Moorhouse AJ Nabekura J. Microglia and synapse interactions: fine tuning neural circuits and candidate molecules. Front Cell Neurosci. (2013) 7:70. 10.3389/fncel.2013.00070 23720611 PMC3654203

[13] Ginhoux F Lim S Hoeffel G Low D Huber T. Origin and differentiation of microglia. Front Cell Neurosci. (2013) 7:45. 10.3389/fncel.2013.00045 23616747 PMC3627983

[14] Ellert-Miklaszewska A Dabrowski M Lipko M Sliwa M Maleszewska M Kaminska B. Molecular definition of the pro-tumorigenic phenotype of glioma-activated microglia. Glia. (2013) 61:1178–90. 10.1002/glia.22510 23650109

[15] Bowman RL Klemm F Akkari L Pyonteck SM Sevenich L Quail DF. Macrophage ontogeny underlies differences in tumor-specific education in brain malignancies. Cell Rep. (2016) 17:2445–59. 10.1016/j.celrep.2016.10.052 27840052 PMC5450644

[16] Prinz M Priller J Sisodia SS Ransohoff RM. Heterogeneity of CNS myeloid cells and their roles in neurodegeneration. Nat Neurosci. (2011) 14:1227–35. 10.1038/nn.2923 21952260

[17] Chen Z Feng X Herting CJ Garcia VA Nie K Pong WW. Cellular and molecular identity of tumor-associated macrophages in glioblastoma. Cancer Res. (2017) 77:2266–78. 10.1158/0008-5472.CAN-16-2310 28235764 PMC5741820

[18] Gieryng A Pszczolkowska D Walentynowicz KA Rajan WD Kaminska B. Immune microenvironment of gliomas. Lab Investig. (2017) 97:498–518. 10.1038/labinvest.2017.19 28287634

[19] Ellert-Miklaszewska A Wisniewski P Kijewska M Gajdanowicz P Pszczolkowska D Przanowski P. Tumour-processed osteopontin and lactadherin drive the protumorigenic reprogramming of microglia and glioma progression. Oncogene. (2016) 35:6366–77. 10.1038/onc.2016.55 27041573

[20] Hu F Dzaye O Hahn A Yu Y Scavetta RJ Dittmar G. Glioma-derived versican promotes tumor expansion via glioma-associated microglial/macrophages Toll-like receptor 2 signaling. Neuro Oncol. (2015) 17:200–10. 10.1093/neuonc/nou324 25452390 PMC4288527

[21] Zhou W Ke SQ Huang Z Flavahan W Fang X Paul J. Periostin secreted by glioblastoma stem cells recruits M2 tumour-associated macrophages and promotes malignant growth. Nat Cell Biol. (2015) 17:170–82. 10.1038/ncb3090 25580734 PMC4312504

[22] Zhang M Olsson Y. Hematogenous metastases of the human brain–characteristics of peritumoral brain changes: a review. J Neurooncol. (1997) 35:81–9. 10.1023/A:1005799805335 9266444

[23] Hambardzumyan D Gutmann DH Kettenmann H. The role of microglia and macrophages in glioma maintenance and progression. Nat Neurosci. (2015) 19:20–7. 10.1038/nn.4185 26713745 PMC4876023

[24] Budczies J von Winterfeld M Klauschen F Bockmayr M Lennerz JK Denkert C. The landscape of metastatic progression patterns across major human cancers. Oncotarget. (2015) 6:570–83. 10.18632/oncotarget.2677 25402435 PMC4381616

[25] Schouten LJ Rutten J Huveneers HAM Twijnstra A. Incidence of brain metastases in a cohort of patients with carcinoma of the breast, colon, kidney, and lung and melanoma. Cancer. (2002) 94:2698–705. 10.1002/cncr.10541 12173339

[26] Barnholtz-Sloan JS Sloan AE Davis FG Vigneau FD Lai P Sawaya RE. Incidence proportions of brain metastases in patients diagnosed (1973 to 2001) in the metropolitan detroit cancer surveillance system. J Clin Oncol. (2004) 22:2865–72. 10.1200/JCO.2004.12.149 15254054

[27] Cagney DN Martin AM Catalano PJ Redig AJ Lin NU Lee EQ. Incidence and prognosis of patients with brain metastases at diagnosis of systemic malignancy: a population-based study. Neuro Oncol. (2017) 19:1511–21. 10.1093/neuonc/nox077 28444227 PMC5737512

[28] Tsukada Y Fouad A Pickren JW Lane WW. Central nervous system metastasis from breast carcinoma. Autopsy study. Cancer. (1983) 52:2349–54. 6640506 10.1002/1097-0142(19831215)52:12<2349::aid-cncr2820521231>3.0.co;2-b

[29] Giordano SH Buzdar AU Smith TL Kau SW Yang Y Hortobagyi GN Is breast cancer survival improving? trends in survival for patients with recurrent breast cancer diagnosed from 1974 through 2000. Cancer. (2004) 100:44–52. 10.1002/cncr.11859 14692023

[30] Tabouret E Chinot O Metellus P Tallet A Viens P Gonçalves A. Recent trends in epidemiology of brain metastases: an overview. Anticancer Res. (2012) 32:4655–62. 23155227

[31] Von Hoff DD Ervin T Arena FP Chiorean EG Infante J Moore M. Increased survival in pancreatic cancer with nab-paclitaxel plus gemcitabine. N Engl J Med. (2013) 369:1691–703. 10.1056/NEJMoa1304369 24131140 PMC4631139

[32] Abraham J Abiraterone increases survival in metastatic prostate cancer. Community Oncol. (2012) 9:240–2. 10.1016/j.cmonc.2012.07.002

[33] Chaffer CL Weinberg RA. A perspective on cancer cell metastasis. Science. (2011) 331:1559–64. 10.1126/science.1203543 21436443

[34] Stelzer K. Epidemiology and prognosis of brain metastases. Surg Neurol Int. (2013) 4(Suppl. 4):S192–202. 10.4103/2152-7806.111296 23717790 PMC3656565

[35] Hatiboglu MA Chang EL Suki D Sawaya R Wildrick DM Weinberg JS. Outcomes and prognostic factors for patients with brainstem metastases undergoing stereotactic radiosurgery. Neurosurgery. (2011) 69:796–806. 10.1227/NEU.0b013e31821d31de 21508879

[36] Taggart D Andreou T Scott KJ Williams J Rippaus N Brownlie RJ. Anti-PD-1/anti-CTLA-4 efficacy in melanoma brain metastases depends on extracranial disease and augmentation of CD8+ T cell trafficking. Proc Natl Acad Sci USA. (2018) 115:E1540–9. 10.1073/pnas.1714089115 29386395 PMC5816160

[37] Iorgulescu JB Harary M Zogg CK Ligon KL Reardon DA Hodi FS. Improved risk-adjusted survival for melanoma brain metastases in the era of checkpoint blockade immunotherapies: results from a national cohort. Cancer Immunol Res. (2018) 6:1039–45. 10.1158/2326-6066.CIR-18-0067 30002157 PMC6230261

[38] Sloot S Chen YA Zhao X Weber JL Benedict JJ Mulé JJ. Improved survival of patients with melanoma brain metastases in the era of targeted BRAF and immune checkpoint therapies. Cancer. (2018) 124:297–305. 10.1002/cncr.30946 29023643 PMC7771556

[39] Ahmed KA Stallworth DG Kim Y Johnstone PAS Harrison LB Caudell JJ. Clinical outcomes of melanoma brain metastases treated with stereotactic radiation and anti-PD-1 therapy. Ann Oncol. (2016) 27:434–41. 10.1093/annonc/mdv622 26712903

[40] Graus F Walker RW Allen JC. Brain metastases in children. J Pediatr. (1983) 103:558–61. 10.1016/S0022-3476(83)80583-6 6620015

[41] Kebudi R Ayan I Görgün Ö Agaoglu FY Vural S Darendeliler E. Brain metastasis in pediatric extracranial solid tumors: survey and literature review. J Neurooncol. (2005) 71:43–8. 10.1007/s11060-004-4840-y 15719274

[42] Dolgushin M Kornienko V Pronin I Brain Metastases: Advanced Neuroimaging. Cham: Springer International Publishing (2017).

[43] Chen F Zhang Y Varambally S Creighton CJ. Molecular correlates of metastasis by systematic pan-cancer analysis across the cancer genome atlas. Mol Cancer Res. (2018) 17:476–87. 10.1158/1541-7786.MCR-18-0601 30401717 PMC6359982

[44] Zehir A Benayed R Shah RH Syed A Middha S Kim HR Mutational landscape of metastatic cancer revealed from prospective clinical sequencing of 10,000 patients. Nat Med. (2017) 23:703–13. 10.1038/nm.4333 28481359 PMC5461196

[45] Berghoff AS Lassmann H Preusser M Höftberger R. Characterization of the inflammatory response to solid cancer metastases in the human brain. Clin Exp Metastasis. (2013) 30:69–81. 10.1007/s10585-012-9510-4 22752508

[46] Sperduto PW Kased N Roberge D Xu Z Shanley R Luo X. Summary report on the graded prognostic assessment: an accurate and facile diagnosis-specific tool to estimate survival for patients with brain metastases. J Clin Oncol. (2012) 30:419–25. 10.1200/JCO.2011.38.0527 22203767 PMC3269967

[47] Chen AM Jahan TM Jablons DM Garcia J Larson DA. Risk of cerebral metastases and neurological death after pathological complete response to neoadjuvant therapy for locally advanced nonsmall-cell lung cancer: clinical implications for the subsequent management of the brain. Cancer. (2007) 109:1668–75. 10.1002/cncr.22565 17342770

[48] Levy A Faivre-Finn C Hasan B De Maio E Berghoff AS Girard N. Diversity of brain metastases screening and management in non-small cell lung cancer in Europe: results of the European Organisation for Research and Treatment of Cancer Lung Cancer Group survey. Eur J Cancer. (2018) 93:37–46. 10.1016/j.ejca.2018.01.067 29477100

[49] Slotman B Faivre-Finn C Kramer G Rankin E Snee M Hatton M. Prophylactic cranial irradiation in extensive small-cell lung cancer. N Engl J Med. (2007) 357:664–72. 10.1056/NEJMoa071780 17699816

[50] Horn L Mansfield AS Szczesna A Havel L Krzakowski M Hochmair MJ. First-line atezolizumab plus chemotherapy in extensive-stage small-cell lung cancer. N Engl J Med. (2018) 379:2220–9. 10.1056/NEJMoa1809064 30280641

[51] Ge M Zhuang Y Zhou X Huang R Liang X Zhan Q. High probability and frequency of EGFR mutations in non-small cell lung cancer with brain metastases. J Neurooncol. (2017) 135:413–8. 10.1007/s11060-017-2590-x 28780743

[52] Soria JC Ohe Y Vansteenkiste J Reungwetwattana T Chewaskulyong B Lee KH. Osimertinib in untreated EGFR -mutated advanced non–small-cell lung cancer. N Engl J Med. (2017) 378:113–25. 10.1056/NEJMoa1713137 29151359

[53] Reck M Rodríguez-Abreu D Robinson AG Hui R Csoszi T Fülöp A. Pembrolizumab versus chemotherapy for PD-L1–positive non–small-cell lung cancer. N Engl J Med. (2016) 375:1823–33. 10.1056/NEJMoa1606774 27718847

[54] Brahmer J Reckamp KL Baas P Crinò L Eberhardt WEE Poddubskaya E. Nivolumab versus docetaxel in advanced squamous-cell non–small-cell lung cancer. N Engl J Med. (2015) 373:123–35. 10.1056/NEJMoa1504627 26028407 PMC4681400

[55] Kałuza J. The monoclonal antibody (MAB) CD 68 allows the immunocytochemical identification of macrophages in primary and metastatic brain tumors in paraffin embedded tissues. Folia Histochem Cytobiol. (1992) 30:125–7. 1286726

[56] He BP Wang JJ Zhang X Wu Y Wang M Bay BH. Differential reactions of microglia to brain metastasis of lung cancer. Mol Med. (2006) 12:161–70. 10.2119/2006-00033.He 17088948 PMC1626596

[57] Strik HM Stoll M Meyermann R. Immune cell infiltration of intrinsic and metastatic intracranial tumours. Anticancer Res. (2004) 24:37–42. 15015573

[58] Berghoff AS Ricken G Wilhelm D Rajky O Widhalm G Dieckmann K. Tumor infiltrating lymphocytes and PD-L1 expression in brain metastases of small cell lung cancer (SCLC). J Neurooncol. (2016) 130:19–29. 10.1007/s11060-016-2216-8 27436101

[59] Berghoff AS Fuchs E Ricken G Mlecnik B Bindea G Spanberger T. Density of tumor-infiltrating lymphocytes correlates with extent of brain edema and overall survival time in patients with brain metastases. Oncoimmunology. (2016) 5:e1057388. 10.1080/2162402X.2015.1057388 26942067 PMC4760339

[60] El Rassy E Botticella A Kattan J Le Péchoux C Besse B Hendriks L. Non-small cell lung cancer brain metastases and the immune system: from brain metastases development to treatment. Cancer Treat Rev. (2018) 68:69–79. 10.1016/j.ctrv.2018.05.015 29883857

[61] Lin NU Claus E Sohl J Razzak AR Arnaout A Winer EP. Sites of distant recurrence and clinical outcomes in patients with metastatic triple-negative breast cancer: high incidence of central nervous system metastases. Cancer. (2008) 113:2638–45. 10.1002/cncr.23930 18833576 PMC2835546

[62] Leyland-Jones B. Human epidermal growth factor receptor 2-positive breast cancer and central nervous system metastases. J Clin Oncol. (2009) 27:5278–86. 10.1200/JCO.2008.19.8481 19770385

[63] Thein KZ P1-17-08. Efficacy of lapatinib and capecitabine combination therapy in brain metastases from HER-2 positive metastatic breast cancer: a systematic review and meta- analysis. Sabcs. (2017) 78:P1-17-08 10.1158/1538-7445.SABCS17-P1-17-08

[64] Pukrop T Dehghani F Chuang HN Lohaus R Bayanga K Heermann S. Microglia promote colonization of brain tissue by breast cancer cells in a Wnt-dependent way. Glia. (2010) 58:1477–89. 10.1002/glia.21022 20549749

[65] Lorger M Felding-Habermann B. Capturing changes in the brain microenvironment during initial steps of breast cancer brain metastasis. Am J Pathol. (2010) 176:2958–71. 10.2353/ajpath.2010.090838 20382702 PMC2877856

[66] Fitzgerald DP Palmieri D Hua E Hargrave E Herring JM Qian Y. Reactive glia are recruited by highly proliferative brain metastases of breast cancer and promote tumor cell colonization. Clin Exp Metastasis. (2008) 25:799–810. 10.1007/s10585-008-9193-z 18649117 PMC2679391

[67] Zlotnik A Burkhardt AM Homey B. Homeostatic chemokine receptors and organ-specific metastasis. Nat Rev Immunol. (2011) 11:597–606. 10.1038/nri3049 21866172

[68] Chuang HN van Rossum D Sieger D Siam L Klemm F Bleckmann A. Carcinoma cells misuse the host tissue damage response to invade the brain. Glia. (2013) 61:1331–46. 10.1002/glia.22518 23832647 PMC3842117

[69] Rippaus N Taggart D Williams J Andreou T Wurdak H Wronski K. Metastatic site-specific polarization of macrophages in intracranial breast cancer metastases. Oncotarget. (2016) 7:41473–87. 10.18632/oncotarget.9445 27203741 PMC5173073

[70] Andreou KE Soto MS Allen D Economopoulos V de Bernardi A Larkin JR. Anti-inflammatory microglia/macrophages as a potential therapeutic target in brain metastasis. Front Oncol. (2017) 7:251. 10.3389/fonc.2017.00251 29164051 PMC5670100

[71] Liu Y Kosaka A Ikeura M Kohanbash G Fellows-Mayle W Snyder LA. Premetastatic soil and prevention of breast cancer brain metastasis. Neuro Oncol. (2013) 15:891–903. 10.1093/neuonc/not031 23595625 PMC3688013

[72] Qian BZ Li J Zhang H Kitamura T Zhang J Campion LR. CCL2 recruits inflammatory monocytes to facilitate breast-tumour metastasis. Nature. (2011) 475:222–5. 10.1038/nature10138 21654748 PMC3208506

[73] Blazquez R Wlochowitz D Wolff A Seitz S Wachter A Perera-Bel J. PI3K: a master regulator of brain metastasis-promoting macrophages/microglia. Glia. (2018) 66:2438–55. 10.1002/glia.23485 30357946

[74] Hong AM Suo C Valenzuela M Haydu LE Jacobsen KD Reisse CH. Low incidence of melanoma brain metastasis in the hippocampus. Radiother Oncol. (2014) 111:59–62. 10.1016/j.radonc.2014.01.012 24560764

[75] Gummadi T Zhang BY Valpione S Kim C Kottschade LA Mittapalli RK. Impact of BRAF mutation and BRAF inhibition on melanoma brain metastases. Melanoma Res. (2015) 25:75–9. 10.1097/CMR.0000000000000133 25426645

[76] Davies MA Saiag P Robert C Grob JJ Flaherty KT Arance A. Dabrafenib plus trametinib in patients with BRAFV600-mutant melanoma brain metastases (COMBI-MB): a multicentre, multicohort, open-label, phase 2 trial. Lancet Oncol. (2017) 18:863–73. 10.1016/S1470-2045(17)30429-1 28592387 PMC5991615

[77] Pasquali S Chiarion-Sileni V Rossi CR Mocellin S. Immune checkpoint inhibitors and targeted therapies for metastatic melanoma: a network meta-analysis. Cancer Treat Rev. (2017) 54:34–42. 10.1016/j.ctrv.2017.01.006 28189914

[78] Redmer T. Deciphering mechanisms of brain metastasis in melanoma - the gist of the matter. Mol Cancer. (2018) 17:106. 10.1186/s12943-018-0854-5 30053879 PMC6064184

[79] Byers HR Etoh T Lee KW Mihm MC Gattoni-Celli S. Organ-specific metastases in immunodeficient mice injected with human melanoma cells: a quantitative pathological analysis. Melanoma Res. (1993) 3:247–53. 8219757

[80] Zhang C Zhang F Tsan R Fidler IJ Transforming growth factor- 2 is a molecular determinant for site-specific melanoma metastasis in the brain. Cancer Res. (2009) 69:828–35. 10.1158/0008-5472.CAN-08-2588 19141644 PMC2633423

[81] Schwartz H Blacher E Amer M Livneh N Abramovitz L Klein A. Incipient melanoma brain metastases instigate astrogliosis and neuroinflammation. Cancer Res. (2016) 76:4359–71. 10.1158/0008-5472.CAN-16-0485 27261506

[82] Qiao S Qian Y Xu G Luo Q Zhang Z. Long-term characterization of activated microglia/macrophages facilitating the development of experimental brain metastasis through intravital microscopic imaging. J Neuroinflammation. (2019) 16:4. 10.1186/s12974-018-1389-9 30616691 PMC6323850

[83] Nayak L Abrey LE Iwamoto FM. Intracranial dural metastases. Cancer. (2009) 115:1947–53. 10.1002/cncr.24203 19241421

[84] Chamberlain MC Leptomeningeal metastasis. Curr Opin Oncol. (2010) 22:627–35. 10.1097/CCO.0b013e32833de986 20689429

[85] Brower JV Saha S Rosenberg SA Hullett CR Ian Robins H. Management of leptomeningeal metastases: prognostic factors and associated outcomes. J Clin Neurosci. (2016) 27:130–7. 10.1016/j.jocn.2015.11.012 26778048

[86] Le Rhun E Weller M Brandsma D Van den Bent M de Azambuja E Henriksson R. EANO–ESMO clinical practice guidelines for diagnosis, treatment and follow-up of patients with leptomeningeal metastasis from solid tumours. Ann Oncol. (2017) 28:iv84–99. 10.1093/annonc/mdx221 28881917

[87] Goldmann T Wieghofer P Jordão MJC Prutek F Hagemeyer N Frenzel K. Origin, fate and dynamics of macrophages at central nervous system interfaces. Nat Immunol. (2016) 17:797–805. 10.1038/ni.3423 27135602 PMC4968048

[88] Bechmann I Priller J Kovac A Böntert M Wehner T Klett FF. Immune surveillance of mouse brain perivascular spaces by blood-borne macrophages. Eur J Neurosci. (2001) 14:1651–8. 10.1046/j.0953-816x.2001.01793.x 11860459

[89] Grier J Batchelor T. Metastatic neurologic complications of non-Hodgkin's lymphoma. Curr Oncol Rep. (2005) 7:55–60. 10.1007/s11912-005-0026-9 15610687

[90] Clarke M Gaynon P Hann I Harrison G Masera G Peto R. CNS-directed therapy for childhood acute lymphoblastic leukemia: Childhood ALL Collaborative Group overview of 43 randomized trials. J Clin Oncol. (2003) 21:1798–809. 10.1200/JCO.2003.08.047 12721257

[91] Jost TR Borga C Radaelli E Romagnani A Perruzza L Omodho L. Role of CXCR4-mediated bone marrow colonization in CNS infiltration by T cell acute lymphoblastic leukemia. J Leukoc Biol. (2016) 99:1077–87. 10.1189/jlb.5MA0915-394R 26931577

[92] Yao H Price TT Cantelli G Ngo B Warner MJ Olivere L. Leukaemia hijacks a neural mechanism to invade the central nervous system. Nature. (2018) 560:55–60. 10.1038/s41586-018-0342-5 30022166 PMC10257142

[93] Fukumura K Kawazu M Kojima S Ueno T Sai E Soda M. Genomic characterization of primary central nervous system lymphoma. Acta Neuropathol. (2016) 131:865–75. 10.1007/s00401-016-1536-2 26757737

[94] Kasenda B Ferreri AJM Marturano E Forst D Bromberg J Ghesquieres H. First-line treatment and outcome of elderly patients with primary central nervous system lymphoma (PCNSL)-a systematic review and individual patient data meta-analysis. Ann Oncol. (2015) 26:1305–13. 10.1093/annonc/mdv076 25701456 PMC4735103

[95] Nakamura T Yamashita S Fukumura K Nakabayashi J Tanaka K Tamura K. Genome-wide DNA methylation profiling identifies primary central nervous system lymphoma as a distinct entity different from systemic diffuse large B-cell lymphoma. Acta Neuropathol. (2017) 133:321–4. 10.1007/s00401-016-1664-8 28058506

[96] Komohara Y Horlad H Ohnishi K Ohta K Makino K Hondo H. M2 macrophage/microglial cells induce activation of Stat3 in primary central nervous system lymphoma. J Clin Exp Hematop. (2011) 51:93–9. 10.3960/jslrt.51.93 22104307

[97] Sasayama T Tanaka K Mizowaki T Nagashima H Nakamizo S Tanaka H. Tumor-associated macrophages associate with cerebrospinal fluid interleukin-10 and survival in primary central nervous system lymphoma (PCNSL). Brain Pathol. (2016) 26:479–87. 10.1111/bpa.12318 26314692 PMC8029390

[98] Sasayama T Nakamizo S Nishihara M Kawamura A Tanaka H Mizukawa K. Cerebrospinal fluid interleukin-10 is a potentially useful biomarker in immunocompetent primary central nervous system lymphoma (PCNSL). Neuro Oncol. (2012) 14:368–80. 10.1093/neuonc/nor203 22156547 PMC3280797

[99] Song Y Zhang W Zhang L Wu W Zhang Y Han X. Cerebrospinal fluid IL-10 and IL-10/IL-6 as accurate diagnostic biomarkers for primary central nervous system large B-cell lymphoma. Sci Rep. (2016) 6:38671. 10.1038/srep38671 27924864 PMC5141427

[100] Nguyen-Them L Costopoulos M Tanguy ML Houillier C Choquet S Benanni H. The CSF IL-10 concentration is an effective diagnostic marker in immunocompetent primary CNS lymphoma and a potential prognostic biomarker in treatment-responsive patients. Eur J Cancer. (2016) 61:69–76. 10.1016/j.ejca.2016.03.080 27156226

[101] Nam SJ Kim S Kwon D Kim H Kim S Lee E. Prognostic implications of tumor-infiltrating macrophages, M2 macrophages, regulatory T-cells, and indoleamine 2,3-dioxygenase-positive cells in primary diffuse large B-cell lymphoma of the central nervous system. Oncoimmunology. (2018) 7:e1442164. 10.1080/2162402X.2018.1442164 29900049 PMC5993494

[102] Yushi Q Li Z Von Roemeling CA Doeppler H Marlow LA Kim BYS. Osteopontin is a multi-faceted pro-tumorigenic driver for central nervous system lymphoma. Oncotarget. (2016) 7:32156–71. 10.18632/oncotarget.8537 27050077 PMC5078004

[103] Takahashi Y Sawada T Akahane T Kawase Y Ikeda H Makino K. Monocyte chemoattractant protein 1 expression and proliferation in primary central nervous system lymphoma. Oncol Lett. (2017) 14:264–70. 10.3892/ol.2017.6122 28693163 PMC5494900

[104] Rubenstein JL Shen A Batchelor TT Kadoch C Treseler P Shuman MA. Differential gene expression in central nervous system lymphoma. Blood. (2009) 113:266–7. 10.1182/blood-2008-04-152835 19122120 PMC2614638

[105] Montesinos-Rongen M Sanchez-Ruiz M Brunn A Hong K Bens S Perales SR. Mechanisms of intracerebral lymphoma growth delineated in a syngeneic mouse model of central nervous system lymphoma. J Neuropathol Exp Neurol. (2013) 72:325–36. 10.1097/NEN.0b013e31828b7a98 23481709

[106] Chapuy B Roemer MGM Stewart C Tan Y Abo RP Zhang L. Targetable genetic features of primary testicular and primary central nervous system lymphomas. Blood. (2016) 127:869–81. 10.1182/blood-2015-10-673236 26702065 PMC4760091

[107] Nayak L Iwamoto FM Lacasce A Mukundan S Roemer MGM Chapuy B. PD-1 blockade with nivolumab in relapsed/refractory primary central nervous system and testicular lymphoma. Blood. (2017) 129:3071–3. 10.1182/blood-2017-01-764209 28356247 PMC5766844

[108] Hayano A Komohara Y Takashima Y Takeya H Homma J Fukai J. Programmed cell death ligand 1 expression in primary central nervous system lymphomas: a clinicopathological study. Anticancer Res. (2017) 37:5655–66. 10.21873/anticanres.12001 28982883

[109] Raza M Prasad P Gupta P Kumar N Sharma T Rana M. Perspectives on the role of brain cellular players in cancer-associated brain metastasis: translational approach to understand molecular mechanism of tumor progression. Cancer Metastasis Rev. (2018) 37:791–804. 10.1007/s10555-018-9766-5 30284650

